# Psychiatric manifestations of Susac Syndrome: a case report

**DOI:** 10.1192/j.eurpsy.2023.2113

**Published:** 2023-07-19

**Authors:** M. Gomes, P. Veloso, E. Monteiro, R. Faria, E. Freitas

**Affiliations:** 1 Psychiatry; 2 Neurology, Hospital de Braga, Braga, Portugal

## Abstract

**Introduction:**

Susac Syndrome (SS) is an immune-mediated endotheliopathy that mainly affects young women. It is characterized by the typical triad: subacute encephalopathy, retinal vaso-occlusive disease, and hearing loss. Encephalopathy symptoms are varied and include memory loss, psychiatric disturbances, cranial nerve disorders, seizures, and dementia. The syndrome is considered a rare but important differential diagnosis in various neurological, psychiatric, ophthalmological, and ear-nose-throat disorders.

**Objectives:**

Report a clinical case of SS to reflect on the relationship between psychiatric and neurological symptoms and on immune-mediated psychiatric symptoms.

**Methods:**

Collection of clinical information from the patient described below. Review of the literature about SS.

**Results:**

A 21-year-old woman presented to the Psychiatry Emergency Department in November 2021 for complaints of sadness, anhedonia and emotional lability, with one month of evolution. She also had insomnia and confusional periods, so she stopped driving and quit her work as a storekeeper. She was given sertraline 50mg/day and trazodone 50mg/day. In the past two weeks, the patient had episodes of headache and vomiting, with 8 kg weight loss. She started a fever (38.5ºC) two days before observation. The patient had a prior history of depressive symptomatology four years earlier related to her father’s grief and her medical and surgical history was unremarkable. She was brought in a wheelchair by her mother and was using diapers because she was confined to bed for the past week. Objectively, the patient was somnolent, tearful and confused, with scarce speech and psychomotor slowing. No focal signs were found on neurological examination. Collaboration of Neurology was asked. Routine laboratory studies showed a slight increase in leucocyte count (12 500/mm3) and CRP (17mg/dL). Cerebrospinal fluid analysis showed 15 cells/uL and protein of 2.64 g/L. Cerebral MRI showed multiple striatocapsular periventricular lesions involving the thalamus, the left midbrain, and the medulla oblongata, as well as focal bilateral hemispherical and cerebellar subcortical lesions. The lesions presented high signal in T2 and showed restriction in the diffusion study. She was admitted to the Neurology inpatient department and treated with pulse methylprednisolone 1000 mg/day for five consecutive days, after which cognitive function improved. Ophthalmology observation found cotton-wool exudate and arteriolar interruption in the right eye, supporting the diagnosis of SS.

**Conclusions:**

This syndrome represents the importance of diligent cooperation among different medical specialties to improve diagnosis-making, treatment and recovery. Psychiatric symptoms are frequent in neurological syndromes, so a high degree of suspicion is needed.

**Disclosure of Interest:**

None Declared

